# An Assessment of the Factors Influencing the Prediction Accuracy of Genomic Prediction Models Across Multiple Environments

**DOI:** 10.3389/fgene.2021.689319

**Published:** 2021-07-23

**Authors:** Sarah Widener, George Graef, Alexander E. Lipka, Diego Jarquin

**Affiliations:** ^1^Department of Crop Sciences, University of Illinois, Urbana, IL, United States; ^2^Department of Agronomy and Horticulture, University of Nebraska, Lincoln, NE, United States

**Keywords:** genotype-by-environment (GE) interaction, soybean nested association mapping (SoyNAM) populations, genomic selection (GS), extreme environmental conditions, environmental covariates (ECs)

## Abstract

The effects of climate change create formidable challenges for breeders striving to produce sufficient food quantities in rapidly changing environments. It is therefore critical to investigate the ability of multi-environment genomic prediction (GP) models to predict genomic estimated breeding values (GEBVs) in extreme environments. Exploration of the impact of training set composition on the accuracy of such GEBVs is also essential. Accordingly, we examined the influence of the number of training environments and the use of environmental covariates (ECs) in GS models on four subsets of *n* = 500 lines of the soybean nested association mapping (SoyNAM) panel grown in nine environments in the US-North Central Region. The ensuing analyses provided insights into the influence of both of these factors for predicting grain yield in the most and the least extreme of these environments. We found that only a subset of the available environments was needed to obtain the highest observed prediction accuracies. The inclusion of ECs in the GP model did not substantially increase prediction accuracies relative to competing models, and instead more often resulted in negative prediction accuracies. Combined with the overall low prediction accuracies for grain yield in the most extreme environment, our findings highlight weaknesses in current GP approaches for prediction in extreme environments, and point to specific areas on which to focus future research efforts.

## Introduction

The impacts of climate change are adversely affecting the availability of food, feed, fuel, and fiber security worldwide, with prior research suggesting a crop yield loss of 5% for each degree Celsius above historically observed weather patterns (Nelson et al., 2010; Zhao et al., 2017). Accelerated climate change has already been observed in specific regions that have low food security, which in turn could exacerbate crises in areas of the world that already struggle with a lack of available and affordable food (Whitford et al., 2013). It is therefore critical that research efforts focus on refining breeding tools so that the overall genetic gain of crops that humanity relies on continues to increase, even in the face of extreme and fluctuating environments. Such work in breeding for optimal crop varieties are essential because agricultural efficiency in use of land and inputs are maximised whenever growers select the best crop variety for their environment, whereas varieties ill-suited to their environment will be more susceptible to disease, pest, and weather events (Zhao et al., 2017).

Genomic prediction (GP) is an emergent methodology that revolutionised plant and animal breeding, and is grounded in a statistical framework that uses genome-wide markers to predict breeding values of agronomically important traits (Bernardo, 1994; Meuwissen et al., 2001). Bernardo (1994) was the first who proposed the use of genomic information as covariates for predicting untested genotypes. Later on, Meuwissen et al. (2001) proposed a new methodology to cope with the challenge of fitting prediction models when the number of genomic covariates (*p*), delivered with the advancements of sequencing technologies, surpass by far the number data points (*n*) available to fit models (*p* ≫ *n*).

A typical breeding program using GP begins with model training in which individual plants, grouped in a training population, are genotyped and phenotyped for the trait(s) of interest (Heffner et al., 2009). These training data are then used to fit a prediction model that quantifies the relationship between the *p* genotyped markers and phenotypic traits. This fitted model exclusively uses genotypic information collected from a breeding population to predict genomic estimated breeding values (GEBVs) of un-phenotyped genotypes, leveraging the genomic relationships between individuals in testing and training sets. The main application of this fitted GP model is to find which individuals have optimal GEBVs. Arguably, the most important advantages of GP are that it allows breeders (1) to determine which varieties should be discarded (screening), (2) to identify superior individuals to advance, and (3) to select best parents with desirable characteristics to be used in the next improvement cycles. In this way, GP has been shown to increase the genetic gain per field season compared to marker-assisted selection approaches that rely on phenotypic selection (Heffner et al., 2010).

One challenge that GP has already been shown to be well-suited for is the prediction of GEBVs across multiple environments (Burgueño et al., 2012; Heslot et al., 2014; Jarquín et al., 2014; Lopez-Cruz et al., 2015). To accurately make such predictions, GP models are typically augmented with additional terms to account for variability attributable to environments and their interaction with the genotype. These augmented GP models take on two main forms, specifically naïve or non-informed and informed. The first, naïve or non-informed, is to include a main random effect for the environment, as well as a two-way interaction effect between each marker genotype and each environment. This so-called G × E model has been shown to improve prediction accuracies (Jarquín et al., 2014; Lopez-Cruz et al., 2015) relative to conventional GP models that only include genotype and environment main effects. The second approach (informed) takes into account environmental covariates (ECs) measured at each environment, and then uses kernel-based methods to incorporate such information via the variance-covariance structures, which ultimately account for the interaction between environmental factors and marker genotypes. The resulting model (called the G × W model) incorporates quantifications of the interactions between each marker genotype and each EC into the prediction of GEBVs, and it could potentially outperform the naive G × E models (Jarquín et al., 2014; Basnet et al., 2019).

Given the promising prediction accuracies of the G × E and G × W models reported in these previous studies (Basnet et al., 2019), it is critical that their potential to predict GEBVs in extreme environments are explored. If these two models end up yielding a low or similar prediction accuracies under extreme environmental conditions, then future research will need to focus on either refining these GP models, exploring the genetic and environmental diversity required to yield decent prediction accuracies, or both. Therefore, the purpose of this study was to explore the impact of training set composition on the ability of the G × E and G × W models to accurately predict GEBVs in an extreme environment. The resulting analysis was conducted using a subset of the publicly available genotypic, phenotypic, and EC data from the soybean nested association mapping (SoyNAM) panel (Song et al., 2017; Diers et al., 2018) collected across multiple years and locations across the US-North Central Region. We used the phenotypic and EC data available at the nine resulting environments to determine which of the nine resulting environments were most and least similar among them. We then explored which subsets of environments yielded the highest prediction accuracies in these two targeted environments. Our working hypothesis was that the currently available genotypic, phenotypic, and EC data were insufficient for enabling the G × E and G × W GP models to accurately predict GEBVs in extreme environments. Thus, we predicted that these two GP models would provide inaccurate GEBVs at the most different of the nine environments that we considered in this study.

## Materials and Methods

The SoyNAM panel has been previously described (Song et al., 2017). Briefly, this panel consists of 40 recombinant inbred line (RIL) families derived from crossing a diverse parent to a common parent (IA3023). On average, each family consists of approximately 140 RILs, resulting in a total sample size of 5,600 individuals. To conduct our analysis, we considered a total of 5,000 markers that were genotyped from 17 lines that are elite public germplasm; 15 have diverse ancestry and 8 are plant introductions (Xavier et al., 2015). Genotypic and phenotypic data for the SoyNAM are publicly available at https://www.soybase.org/SoyNAM. These markers were then filtered to remove all markers that contain more than 50% of missing values and a minor allele frequency smaller than 0.03, resulting in a total of 4,450 markers being used for all downstream analyses.

### Phenotypic Data and Field Trials

The phenotypic data were collected across 10 different locations in the US-North Central Region over 3 years. The trait that we analysed was grain yield (kg ha^–1^), which has been previously described (Hunter et al., 2017). The experimental design at each of the resulting environments have already been presented in Diers et al. (2018) and Xavier et al. (2018). However, not all locations were observed in all years, which resulted in a total of 18 location × year combinations (environments) (Xavier et al., 2018). Of these 18 environments, we analysed only a subset of 9 environments for which (1) we were able to obtain weather information and (2) have a common set of overlapping genotypes. Thus, a total of 2,336 genotypes were observed across all 9 environments. At each of the 9 environments, best linear unbiased predictions (BLUPs) grain yield, which already have been presented in Diers et al. (2018) and Xavier et al. (2018), were used in our analyses. To ensure the most and the least similar environments based on weather data also were the most and the least similar environments based on phenotypic data, random samples of 500 individuals were selected and mean phenotypic correlation between environments was computed until these matched. For a given environment, the mean phenotypic correlation is defined as the mean Pearson correlation of grain yield and the grain yield at the remaining environments. Thus, four random samples were considered for this study, where the difference of the mean phenotypic Pearson correlation between the most and the least correlated environment ranged from 0.185, 0.190, 0.191 to 0.198. Within each environment, heritabilities for grain yield were estimated as the ratio between the variability explained by the genetic component and the total variance H^2=σ^L2σ^L2+σ^E2, where ${\hat{\sigma }}_{L}^{2}$ and ${\hat{\sigma }}_{E}^{2}$ are, respectively, the variance component estimates of a line random effect and residual random effect fitted from a mixed linear model with grain yield as the response variable and lines included as a random effects (please see Holland et al., 2003) for an overview of calculating heritability).

### Weather Data

At each of the 9 environments, we obtained ECs in the form of historical weather data extrapolated from Google Cloud.^1^ These data were from weather stations distanced at most 57 km from the field location. After downloading the data from the cloud using a custom R script (Available from GitHub^2^), we selected three ECs that were both common to all 9 weather stations and recorded in 24-hr increments. Specifically, these three ECs were mean minimum daily temperature (measured in tenths of degrees Celsius), mean maximum daily temperature (tenths of degrees Celsius), and mean daily precipitation (inches). We chose to not convert mean daily precipitation to SI units because we wanted to leave the historical weather data from Google Cloud unaltered. For each location, weather data were collected starting on the planting date and continued until the 125th day after planting. Thus, the total number of ECs totaled 3 × 125 = 375, for a total of 9 × 375 = 3,375 total weather records across all 9 locations. A total of six weather records were missing; these values were imputed with the mean value between the previous and the following day within the same environment.

### Statistical Analyses Conducted on ECs to Quantify Similarity Across Environments

At each environment, we assessed the distribution of the values of each EC on the first day of planting and the following 125 days. Additionally, we conducted a principal component analysis (Morrison et al., 1976) of all 375 ECs (3 ECs measured across 125 days) to explore their degree of similarity across the 9 environments. These analyses enabled the identification of which environments were most and least similar among them.

### GS Models

We considered three genomic selection models (M1–M3) in our analysis; however, first we introduce the most elemental linear predictor (M0) because it is useful for deriving the other models.

M0: E + L. Consider that the yield performance *y*_*ij*_ of the *i*th (*i* = 1, 2,…, 500) genotype observed in the *j*th (*j* = 1, 2,…, 9) environment can be represented as a sum of a common constant effect across genotypes and environments (μ) plus a line effect *L_i_*, an environmental effect *E_j_* and an error term *e*_*ij*_ addressing the non-explained phenotypic variability as follows in M0:

(0) $$y_{ij}=\mu +E_{j}+L_{i}+e_{ij}$$

where *E_j_* and *L_i_* are considered random terms such that these are assumed to be independent and identically distributed (IID) outcomes from a normal density such that $E_{j}∼\text{N}\left(0,\sigma_{E}^{2}\right)$ and $L_{j}∼\text{N}\left(0,\sigma_{L}^{2}\right)$, with $\sigma_{E}^{2}$ and $\sigma_{L}^{2}$ being the corresponding variance components; and *e*_*ij*_ ∼ N(0, σ^2^) with σ^2^ representing the residual variance. One disadvantage of M0 is that it does not allow the prediction of unobserved genotypes because it relies only on phenotypic information.

M1: E + G. To allow the prediction of untested genotypes, genomic relationships between individuals in training and testing sets should be established first. For this, we construct a covariance structure whose entries contain the genomic similarities between pairs of individuals. Assuming that the marker effects *b_k_* in the linear combination involving *p* markers, ${\text{g}}_{i}=\sum_{k=1}^{p}x_{ik}b_{k}$, follows IID normal densities N(0, $\sigma_{b}^{2}$) and using results from the multivariate normal density we have that that the vector of genomic effects **g** = {g_i_} follows a multivariate normal distribution such that $\text{g}∼N\left(0,\text{G}\sigma_{g}^{2}\right)$, where $G=\frac{{\mathrm{XX}}^{\prime }}{p}$, ***X*** is the centered and scaled (by columns) matrix of molecular markers, and $\sigma_{g}^{2}=p\times \sigma_{b}^{2}.$ Thus, we have the following linear predictor for M1:

(1) $$y_{ij}=\mu +E_{j}+{\text{g}}_{i}+e_{ij},$$

where all terms are as previously described. One of the disadvantages of M1 is that it returns the same genomic effect across environments; thus the direct influence of stimuli unique to particular environments are not taken into consideration.

M2: E + G + G × E. To allow estimations of particular genomic effects within environments, Jarquín et al. (2014) proposed a model that conceptually considered the interaction between each molecular marker and each environment. This model is based on the cell-by-cell product between two covariance structures, one for environmental factors using only the identification of the environments and another for genotypes based on the genomic relationship matrix. Thus, the genotype-by-environment interaction effects can be predicted thought **gE** = {gE_*ij*_} with $\text{gE}∼N\left(0,Z_{E}Z_{E}^{{}^{\prime }}\#Z_{\text{g}}\text{G}Z_{\text{g}}^{{}^{\prime }}\sigma_{\text{gE}}^{2}\right)$ where *Z_E_* and Z_g_ are the corresponding incidence matrices that connect phenotypic observations with environments and genotypes; $\sigma_{\text{gE}}^{2}$ is the corresponding variance component; and # represents the Hadamard (cell-by-cell) product between two matrices. Hence, we have that the resulting linear predictor for M2 can be written as follows:

(2) $$y_{ij}=\mu +E_{j}+{\text{g}}_{i}+\text{g}E_{ij}+e_{ij},$$

where all terms are as previously described. This model not only allows the inclusion of the G × E interaction in a naïve way but potentially also offers the opportunity of including the genotype-by-environment interaction component in an informed way. One approach for accomplishing this is to include ECs that describe environmental similarities between pairs of environments. Such information is incorporated into the final GP model we consider, as described below.

M3: E + G + G × W. Analogous to the derivation of the kinship matrix **G**, the information on ECs can be considered in the development of an environmental kinship matrix Ω describing environmental similarities between pairs of environments. Jarquín et al. (2014) proposed a model that allows the incorporation of the ECs to interact with molecular markers. To accomplish this, it is necessary to first model the main effect of the ECs. Consider that the environmental effect *w_j_* corresponding to *j*^th^ environment can be written as a linear combination between *q* ECs and their corresponding effects $w_{j}=\sum_{l=1}^{q}W_{jl}\gamma_{l}$ with $\gamma_{l}∼N\left(0,\sigma_{W}^{2}\right)$ and $\sigma_{W}^{2}$ defined as the corresponding variance component. Then we have that the vector of environmental effects follows a multivariate normal density such that $\text{w}=\left\{w_{j}\right\}∼N\left(0,\Omega \sigma_{\Omega }^{2}\right)$; where Ω$=\frac{WW^{{}^{\prime }}}{q}$, *W* is the centered and scaled (by columns) matrix of ECs (i.e., measurements of mean minimum daily temperature, mean maximum daily temperature, and mean daily precipitation across 125 days, as previously described), $\sigma_{\Omega }^{2}=q\sigma_{W}^{2}$ is the corresponding variance component. To include the main effect of the ECs in the prediction model, we have to expand Ω using the incidence matrix that connects phenotypes with environments such as $Z_{E}\Omega Z_{E}^{{}^{\prime }}$ is the resulting covariance structure. In order to include the ECs in interaction with marker genotypes, we substitute the expanded covariance matrix in the covariance structure of the **gE** term such as $\text{gw}∼N\left(0,Z_{E}\Omega Z_{E}^{{}^{\prime }}\#Z_{\text{g}}\text{G}Z_{\text{g}}^{{}^{\prime }}\sigma_{g\Omega }^{2}\right)$ with $\sigma_{g\Omega }^{2}$ acting as the corresponding variance component. The resulting linear predictor for M3 can be written as follows:

(3) $$y_{ij}=\mu +E_{j}+{\text{g}}_{i}+\text{g}w_{ij}+e_{ij},$$

where all terms are as previously described. Conceptually, this model allows the inclusion of the interaction between each molecular marker and each ECs.

### Cross-Validation Scheme

The main objective of this cross-validation scheme was to identify the training environments and GP model that yielded the highest possible prediction accuracies in (1) the environment that had the lowest mean phenotypic correlation with the other eight environments, and then to contrast this result with (2) the environment that had the highest mean phenotypic correlation with the other eight environments. For both (1) and (2), we conducted a CV00 cross validation scheme (Jarquín et al., 2017; Jarquin et al., 2020) where none of the genotypes from the test environment were used to train the GS model.

For a given random sample of 500 genotypes (i.e., RILs from the SoyNAM population) observed in all 9 environments, we randomly selected a set of 200 genotypes to be the testing set in the unobserved environment. We used the phenotypic information of the remaining 300 genotypes observed in the remaining 8 environments. Because we were interested in the impact of training set composition on prediction accuracies in a given test environment, we evaluated the ability of all possible subsets of the remaining eight remaining environments to train each GP model and accurately predict GEBVs. The resulting numbers of possible combinations of environments to include in the training set are described in Table 1. For a given test environment, training set, and GP model, prediction accuracy was measured as the Pearson correlation between the observed (phenotypic) and predicted (GEBV) values. This procedure was repeated three additional times so that the performance of the GP models could be evaluated across all 4 random subsets of 500 genotypes.

**TABLE 1:** The number of possible combinations (right column) of subsets of eight environments (left column) considered as a training sets for the genomic selection models.

**Subset of environments**	**Number of combinations**
1	8
2	28
3	56
4	70
5	56
6	28
7	8
8	1

*In summary, the value of the right column of the *i*^*th*^ row is $\left(\begin{array}{c} 8\\ i \end{array}\right)$.*

## Results

### Similar Distribution of EC Values Across Nine Environments, With IA_2013 Displaying the Most Unique EC Values

**FIGURE 1:**
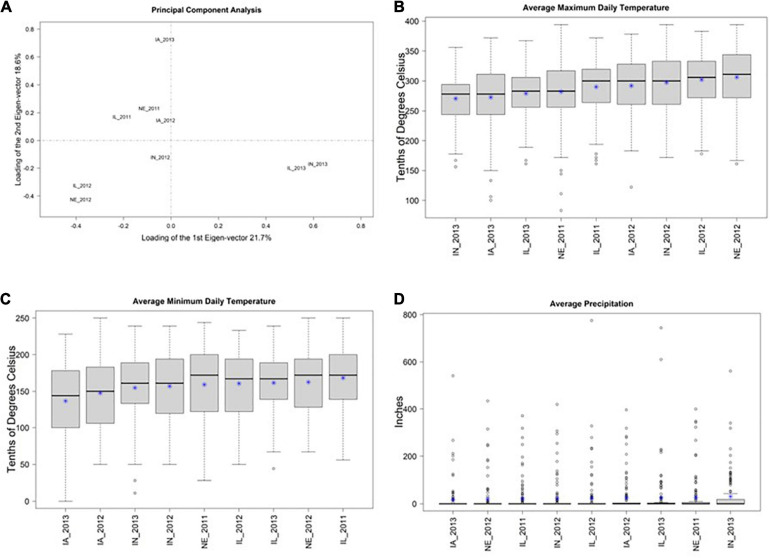
**(A)** Shows the first two principal components (PCs) from a principal component analysis of three environmental covariates (ECs) measured over 125 days at nine environments in the US North Central Region. The *X*-axis shows PC1 and the *Y*-axis shows PC2. The percent of total variation explained in each PC is provided in the axis labels. This plot suggests that IN_2013, IL_2013, and IA_2013 are the environments with EC values that are most distinct from the remaining environments. **(B–D)** Display the distributions of mean daily values of three environmental covariates (*Y*-axis) at these nine environments (*X*-axis) and * represents the mean. **(B,C)** Show maximum and minimum daily temperature, respectively, within each environment in tenths of degrees Celsius. **(D)** Shows precipitation in inches. Collectively **(B–D)** suggest that there the observed EC values are similar among these 9 environments.

A biplot of the first two principal components of 3 × 125 = 375 ECs suggests that many of the locations have similar environmental conditions (Figure 1A). This result is supported by similar distributions of values of the three ECs (across the 125 days since planting) within each of the 9 locations (Figures 1B–D). Collectively, these results suggest that there is not a substantial amount of environmental diversity among the 9 environments that were tested. Nevertheless, among these 9 environments, IL_2013, IN_2013, and IA_2013 appeared to be the most divergent.

### Phenotypic Data on Grain Yield Were Most Unique Within IA_2013, While Grain Yield Within NE_2011 Was Most Similar to the Remaining Environments

**FIGURE 2:**
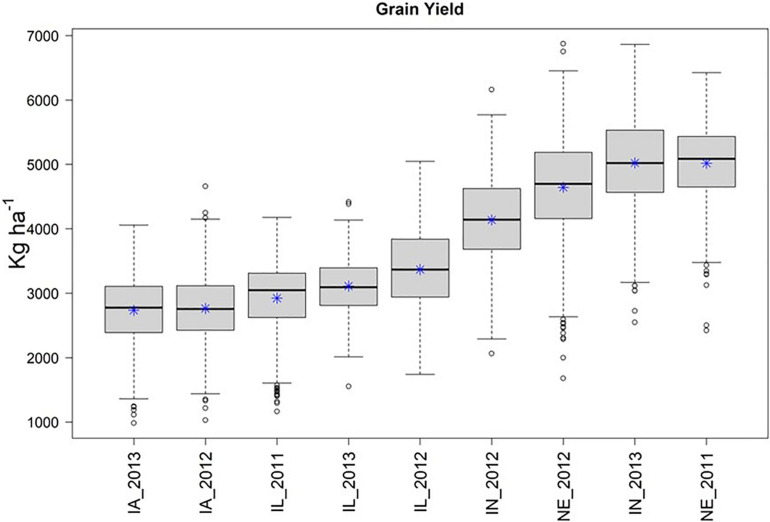
Boxplot of yield in kg ha^–^1 (*Y*-axis), by environment (*X*-axis) for the second random sample of 500 genotypes from the SoyNAM panel, and * represents the sampling mean. Environments IA_2013, IA_2012, and IL_2011 had the lowest yield, while IN_2013 and NE_2011 had the highest yield.

Across the 4 replicate random samples of 500 genotypes, we observed similar trends in phenotypic distributions of yield performance (ka ha^–1^) across the 9 environments. For brevity, we focus on the results for the second random sample in the main text of the manuscript, and then provide similar details for the remaining three random samples in the Supplementary Material section. We observed that IA_2013, IA_2012, and IL_2011 were the environments that tended to yield the least, while IN_2013 and NE_2011 were the environments that yielded the greatest (Figure 2).

We then quantified the phenotypic correlation between environments to determine which were least and most similar. As such, the mean phenotypic correlation of each environment with the remaining eight environments is presented in Table 2. The two environments that showed the lowest and the highest mean correlation with the remaining eight environments were IA_2013 (0.137) and NE_2011 (0.327), respectively (as depicted under the column labeled “Rep 2” under “Average Correlation” in Table 2). Across the four replicates, we also calculated the heritabilities at each of the environments. These heritabilities were relatively stable across the 9 tested environments with around 50% of the phenotypic variability explained by the additive genetic component within each environment (Table 2). Based on the collective information on trait correlations across the 9 environments and the ECs, we determined that IA_2013 was the most extreme environment and that NE_2011 was the least extreme environment.

**TABLE 2:** Mean Pearson correlation coefficient of grain yield (in kg ha^–^1) between each environment and the remaining eight environments (presented under the columns labeled “Average Correlation”), as well as the observed heritability of grain yield within each environment (presented under the columns labeled “Heritability”).

**Environment**	**Average Correlation**				**Heritability**			
	**Rep 1**	**Rep 2**	**Rep 3**	**Rep 4**	**Rep 1**	**Rep 2**	**Rep 3**	**Rep 4**
IA_2013	0.164	0.137	0.150	0.158	0.503	0.485	0.492	0.497
IA_2012	0.268	0.216	0.228	0.254	0.511	0.499	0.483	0.496
IL_2011	0.221	0.203	0.179	0.177	0.500	0.521	0.486	0.514
IL_2013	0.277	0.277	0.257	0.291	0.509	0.510	0.507	0.482
IL_2012	0.263	0.261	0.236	0.247	0.513	0.481	0.497	0.504
IN_2012	0.290	0.277	0.262	0.287	0.507	0.495	0.500	0.483
NE_2012	0.293	0.245	0.235	0.266	0.506	0.484	0.478	0.481
IN_2013	0.289	0.283	0.301	0.276	0.517	0.534	0.502	0.467
NE_2011	0.349	0.327	0.340	0.356	0.502	0.500	0.510	0.499

*The columns labeled “Rep 1”, …, “Rep 4” present these summary statistics for each of the 4 random samples of 500 randomly selected individuals.*

### Relatively Small Number of Environments Needed to Yield Accurate Predictions for IA_2013

**FIGURE 3:**
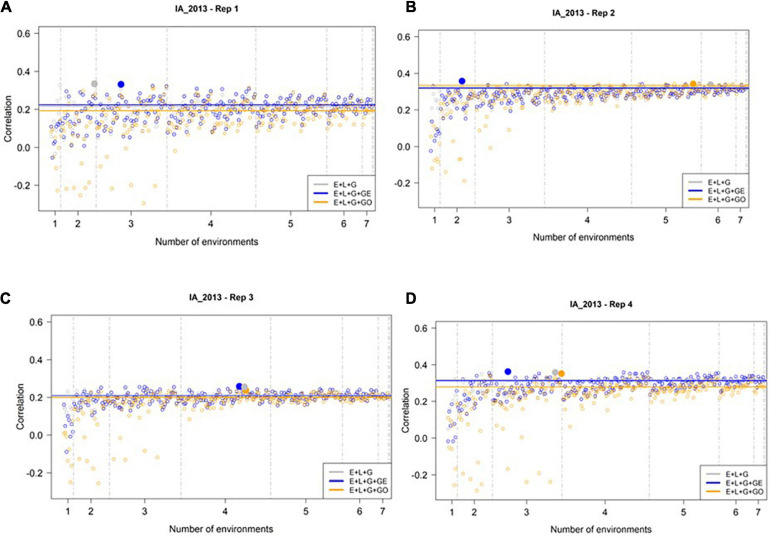
Observed prediction accuracy of grain yield in kg ha^–1^ at IA_2013 across multiple genomic prediction (GP) models and training environments. Four random samples of 500 genotypes from the SoyNAM panel are presented in panels **(A–D)**. For each panel, the *X*-axis is the specific number of environments considered for training the GP model, sorted from smallest to largest number of training environments; and the *Y*-axis shows the prediction accuracy, quantified as the Pearson correlation coefficient between the observed phenotypic values and the genomic estimated breeding values. The results in grey depict the GP model without any genotype-by-environment (G × E) interaction effects, while the results in blue depict the GP model with G × E interaction effects, and finally the results in yellow depict the GP model with G × E interaction effects that incorporates environmental covariates (ECs). The highest observed prediction accuracies across any training set from each GP model are highlighted by a solid circle of the same color, while the prediction accuracies of the three models obtained using all eight of the possible environments in the training set are shown as horizontal lines of the same color. These panels show that not all eight environments are needed to obtain the maximum possible prediction accuracies.

We evaluated the ability of M1–M3 to predict GEBVs in the most extreme environment, IA_2013, using the all-possible subsets of the 8 remaining environments, as described in the Section “Materials and Methods” and Table 1. Figure 3 presents the correlation between the predicted and observed values for IA_2013 considering the 255 different ways for combining the remaining 8 environments for model calibration across the 4 replicates of 500 randomly selected genotypes. In general, low and sometimes negative prediction accuracies were observed, with the highest observed prediction accuracy being less than 0.36. The optimal number of training environments (i.e., that yielded the highest prediction accuracies from M1, M2, and M3) changed considerably across the four replicates, but we frequently observed that a relatively small number of environments was needed to achieve the highest possible prediction accuracy. Across the 4 replicates of 500 random samples, we never observed an instance where the model accounting for ECs (i.e., M3) yielded definitively higher prediction accuracies than M1 or M2. Moreover, there were many combinations of training environments where M3 clearly yielded lower, and often negative, prediction accuracies.

### Slightly Larger Number of Training Environments Needed to Maximize Prediction Accuracy in NE_2011

**FIGURE 4:**
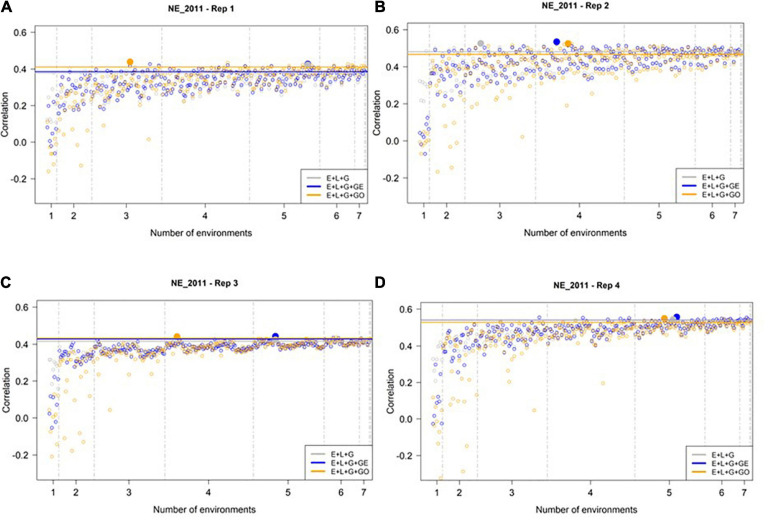
Observed prediction accuracy of grain yield in kg ha^–1^ at NE_2011 across multiple genomic prediction (GP) models and training environments. Four random samples of 500 genotypes from the SoyNAM panel are presented in panels **(A–D)**. For each panel, the *X*-axis is the specific number of environments considered for training the GP model, sorted from smallest to largest number of training environments; and the *Y*-axis shows the prediction accuracy, quantified as the Pearson correlation coefficient between the observed phenotypic values and the genomic estimated breeding values. The results in grey depict the GP model without any genotype-by-environment (G × E) interaction effects, while the results in blue depict the GP model with G × E interaction effects, and finally the results in yellow depict the GP model with G × E interaction effects that incorporates environmental covariates (ECs). The highest observed prediction accuracies across any training set from each GP model are highlighted by a solid circle of the same color, while the prediction accuracies of the three models obtained using all eight of the possible environments in the training set are shown as horizontal lines of the same color. These panels show that not all eight environments are needed to obtain the maximum possible prediction accuracies.

We then conducted a similar analysis to assess the predictive ability of M1–M3 to predict GEBVs in the least extreme environment (NE_2011, see Figure 4). In general, we observed higher prediction accuracies at NE_2011 relative to those observed in the most extreme environment (IA_2013). Similar to IA_2013, the number of optimal environments needed for M1, M2, and M3 differed across reps. However, the general trend we observed was that a larger number of training environments were needed for maximizing the prediction accuracy in NE_2011 relative to IA_2013. Finally, we did not observe any evidence that including ECs in the model improved prediction accuracy. That is, the highest prediction accuracy observed for M3 (∼0.53) was not noticeably different than those of M1 and M2, and the lowest prediction accuracies observed across the four replicates were from M3.

## Discussion

We compared the ability of various subsets of environments to accurately predict GEBVs in (1) a target environment that was the most different from the remaining environments with respect to phenotypic correlation and observed ECs, and (2) a target environment that was the most similar using these same metrics. Although we observed lower prediction accuracies in (1), the ensuing analysis highlighted similar trends in model performance for both (1) and (2). Using three different GS models that accounted for environmental information to varying degrees, we discovered that maximum prediction accuracies could be achieved by using only a subset of the 8 environments to train the GP models. Additionally, we found that the inclusion of ECs into GP models did not substantially boost the prediction accuracies of the target environments. Finally, when using a reduced number of environments to train the GP models, we occasionally observed extremely low and negative prediction accuracies when including ECs into the GP model. Thus, we identified potential areas of weakness in existing GP models when they are applied to predicting GEBVs in specific environments and underscored the critical need to explore which factors influence the development of training environments that can lead to the most accurate of such predictions.

### The Inclusion of ECs Into the GP Model Did Not Result in Substantially Higher Prediction Accuracies

For the environment with the least similar phenotypic correlations and ECs relative to the remaining environments (IA_2013), we observed low prediction accuracies, as expected. These low accuracies indicate that there is room for improvement for developing approaches to predict GEBVs in extreme environments. Nevertheless, the trends that we observed in our analysis point to areas for further exploration and refinement. To illustrate this point, consider the results from the second random sample of 500 individuals we considered in this study. For this replicate of our analysis, the inclusion of the G × E interaction in the model without weather data (M2) returned the highest predictive ability (0.357). In this case, only two environments were needed (IL_2012, IN_2012) for model calibration, and the relative improvements were, respectively, 7, 12, and 10% relative to using all of the eight environments in the training set under M1, M2, and M3.

These results also identified important shortcomings of using ECs directly in the GP model. For instance, the fact that M3 occasionally yielded prediction accuracies that were lower than those of M1 and M2 suggests that the inclusion of ECs into the GP model is not guaranteed to increase the accuracy of GEBVs. This suggests that further research into the development of GP models that effectively incorporate these ECs is needed. Combined with the observation that M3 yielded negative prediction accuracies more often than M1 and M2, we also infer that further investigation similar to Gillberg et al. (2019) is needed into dissecting which EC values are most likely to contribute to the highest possible prediction accuracies. These two avenues for future research could ultimately facilitate the development robust statistical models for GP in this paradigm, as well as identification of the ideal environments and ECs to use to train these GP models.

We observed similar trends between the performance of the three GP models in most similar environment (NE_2011). In particular, we noted that the incorporation of such weather data to predict GEBVs in NE_2011 (i.e., through M3) often resulted in accuracies that were either negative or worse than M1 and M2. Because we observed a higher and more stable prediction accuracies as the number of environments used in the training set increased (a trend that was also observed for IA_2013), we infer that the collective information from multiple similar environments is critical for accurate prediction GEBVs in targeted environments with similar weather characteristics.

### Minimal Genotypic and Environmental Diversity Are Limiting Factors of This Study

There are several important shortcomings of this study. First, we limited our analysis to only one species. Given the relatively narrow genetic diversity of soybean (Hyten et al., 2006), our study potentially did not fully explore the full extent to which M1–M3 could robustly predict breeding values in species with more diverse germplasm. Although we would expect to observe low prediction accuracies for such scenarios (as suggested by the findings of, e.g., Lorenz and Smith, 2015), it would nevertheless be worthwhile to quantify these accuracies. Similarly, all 9 of the environments that we evaluated were from a relatively narrow geographical range in the midwestern United States. Even though we were able to observe differences in the prediction accuracy of the GP models between the two test environments (IA_2013 and NE_2011), it is critical that follow-up studies conduct the analysis presented in this work in data from a wider range of locations.

In general, the incorporation of ECs into GP in a manner analogous to the incorporation of genome-wide marker data is rapidly maturing into the field of enviromics (Resende et al., 2021), and the findings from this study and others (Alves et al., 2021) could be useful for the establishment of best practices for collecting and utilizing environmental data. For example, one notable constraint of our study was that the observed ECs were common for all genotypes within the same environment. Given the potential for significant differences in EC values within a field, we were unable to capture these potentially important sources of variability. Combined with our use of only three ECs that were common across the 9 environments, we postulate that the inclusion of more ECs, potentially with differing values within locations, will reveal how sensitive or insensitive the GP models are at predicting breeding values when used in cases of extreme environments.

## Conclusion

Even with the relatively narrow scope of genomic and environmental diversity observed in our data, we identified notable weaknesses in both the current GP models and training data used to predict GEBVs in different environments. We observed that (1) most accurate GEBVs were from GP models trained on only a subset of the available environments, and (2) at best the inclusion of ECs into the GP model did not substantially improve the prediction accuracies of the GEBVs. Nevertheless, the fact that we observed such diversity in prediction accuracies across the possible combinations of training sets suggest that a substantial amount of research is needed to explore which properties of training sets are responsible for the highest prediction accuracies. Coupled with the generally low prediction accuracies for the most extreme environment, we ultimately conclude that dedicated future research endeavors are needed to make genomic prediction better suited for extreme environments.

## Data Availability Statement

The original contributions presented in the study are included in the article/Supplementary Material, further inquiries can be directed to the corresponding author/s.

## Author Contributions

SW wrote the initial draft, collected weather data, and participated in the conceptualization of the study. GG edited the manuscript and provided guidance to better understand the implementation of GS in soybeans. AL edited the manuscript, provided oversight for the study, and participated in the conceptualization of the study. DJ edited the manuscript, conducted the prediction studies, and participated in the conceptualization of the study. All authors contributed to the article and approved the submitted version.

## Conflict of Interest

The authors declare that the research was conducted in the absence of any commercial or financial relationships that could be construed as a potential conflict of interest.

## Publisher’s Note

All claims expressed in this article are solely those of the authors and do not necessarily represent those of their affiliated organizations, or those of the publisher, the editors and the reviewers. Any product that may be evaluated in this article, or claim that may be made by its manufacturer, is not guaranteed or endorsed by the publisher.

## Footnotes

1 https://cloud.google.com/public-datasets/weather

2 https://github.com/sarahwidener/Frontiers_Paper/blob/master/weather_retrieval/Get%20all%20weather%20data%20for%20project.R

## Supplementary Material

Supplementary Figure 1 Boxplot of yield in kg ha^−1^ (*X* axis), by environment (*Y* axis) for the (first, third or fourth) random sample of 500 genotypes from the SoyNAM panel. Environments IA_2013, IA_2012, and IL_2011 had the lowest yield, while IN_2013 and NE_2011 had the highest yield.

Supplementary Figure 2 Boxplot of yield in kg ha^−1^ (*X* axis), by environment (*Y* axis) for the (first, third or fourth) random sample of 500 genotypes from the SoyNAM panel. Environments IA_2013, IA_2012, and IL_2011 had the lowest yield, while IN_2013 and NE_2011 had the highest yield.

Supplementary Figure 3 Boxplot of yield in kg ha^−1^ (*X* axis), by environment (*Y* axis) for the (first, third or fourth) random sample of 500 genotypes from the SoyNAM panel. Environments IA_2013, IA_2012, and IL_2011 had the lowest yield, while IN_2013 and NE_2011 had the highest yield.
