# Supplementary figures and images for: An Assessment of the Factors Influencing the Prediction Accuracy of Genomic Prediction Models Across Multiple Environments
Source: Front Genet. 2021 Jul 23;12:689319. doi: 10.3389/fgene.2021.689319 (PMC8343134; doi:10.3389/fgene.2021.689319)

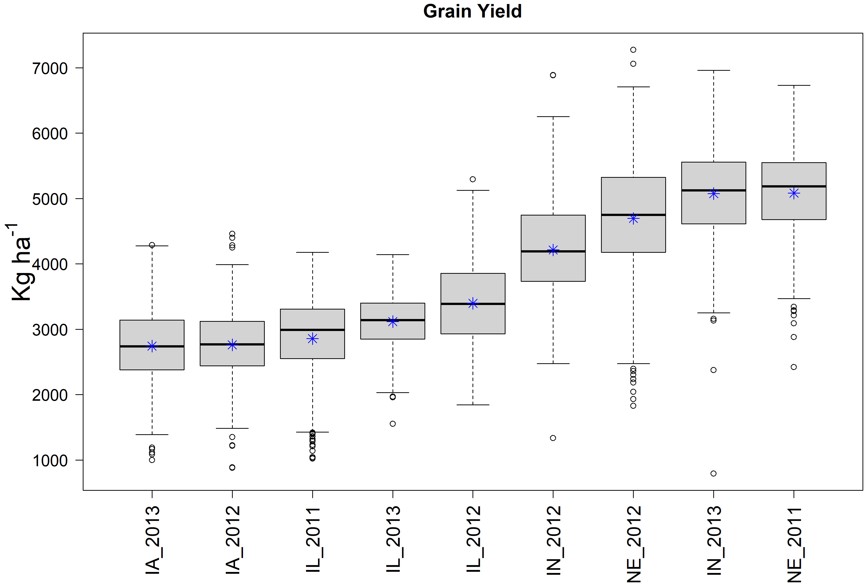

Supplement: Supplementary Figure 1 — Boxplot of yield in kg ha−1 (X axis), by environment (Y axis) for the (first, third or fourth) random sample of 500 genotypes from the SoyNAM panel. Environments IA_2013, IA_2012, and IL_2011 had the lowest yield, while IN_2013 and NE_2011 had the highest yield. [file Image_1.JPEG]

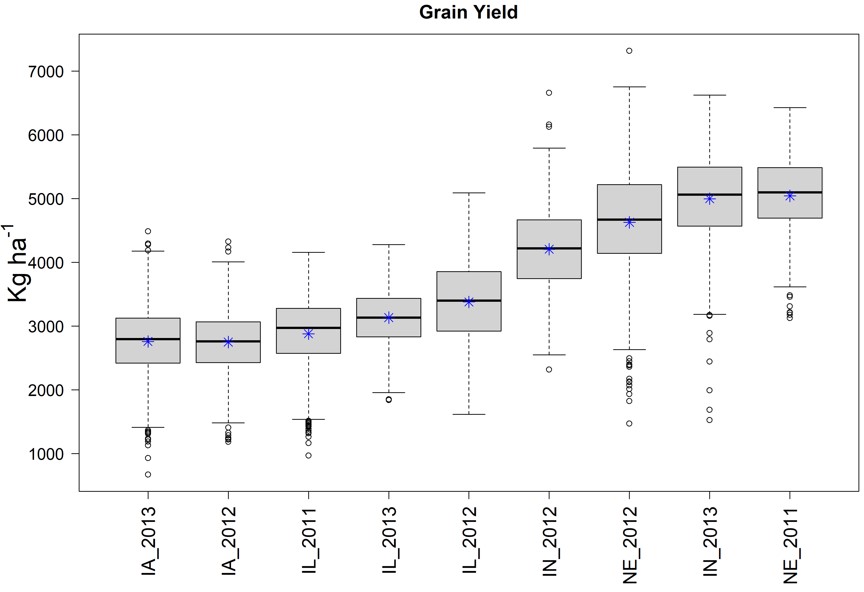

Supplement: Supplementary Figure 2 — Boxplot of yield in kg ha−1 (X axis), by environment (Y axis) for the (first, third or fourth) random sample of 500 genotypes from the SoyNAM panel. Environments IA_2013, IA_2012, and IL_2011 had the lowest yield, while IN_2013 and NE_2011 had the highest yield. [file Image_2.JPEG]

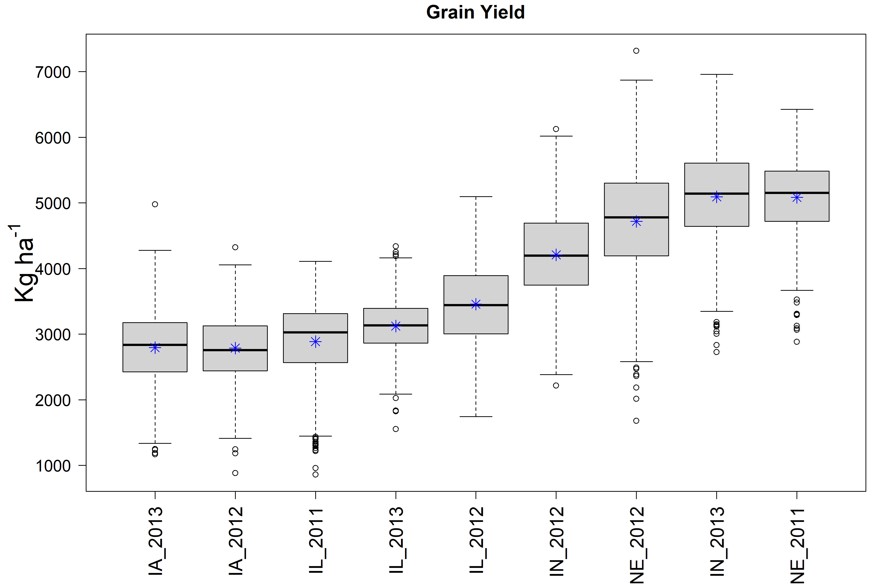

Supplement: Supplementary Figure 3 — Boxplot of yield in kg ha−1 (X axis), by environment (Y axis) for the (first, third or fourth) random sample of 500 genotypes from the SoyNAM panel. Environments IA_2013, IA_2012, and IL_2011 had the lowest yield, while IN_2013 and NE_2011 had the highest yield. [file Image_3.JPEG]
